# Radiological Correlates of Head Injuries in School-Level Rugby Union: A 10-Year Retrospective Cross-Sectional Analysis

**DOI:** 10.1007/s40279-025-02195-5

**Published:** 2025-03-25

**Authors:** Riaan van Tonder, Hofmeyr Viljoen, Christelle Ackermann

**Affiliations:** 1 Division of Radiodiagnosis, Stellenbosch University, Cape Town, South Africa; 2 SCP Radiology, Cape Town, South Africa

## Abstract

**Background:**

Sport-related concussion (SRC) in rugby union is common and carries a high injury burden, especially among children. Computed tomography (CT) imaging is commonly used to assess rugby-related head injuries, including SRC, subjecting children to ionising radiation. In addition, there is concern about the relationship between SRC, repetitive head impacts and neurodegeneration.

**Objective:**

To review and correlate the imaging findings of head injuries in school-level rugby players from a public tertiary referral centre and a private multi-centre radiology service.

**Design:**

Descriptive, retrospective cross-sectional correlational study for the 2014–2023 period.

**Methods:**

Anonymised data were collected from the radiological information systems of a tertiary referral centre and a private radiology provider. Data included participant age, imaging modality, study type, date, findings and SRC status. The public and private datasets were analysed using descriptive and comparative statistics.

**Results:**

A total of 369 cases were identified (public *n* = 132, 36%). Mean participant age was 15 (± 2.5) years, with 78% (*n* = 289) clinically deemed to have an SRC. CT was performed in 347 (94%) cases, with abnormal findings reported in 50 studies (public *n* = 32). The most common findings were craniofacial fractures (*n* = 28) and intracranial injuries (*n* = 19). The sensitivity of CT for detecting SRC was 14%. Public sector participants were more likely to have an SRC (odds ratio: 8.39; 95% CI 8.37–8.41, *p* < 0.001).

**Conclusions:**

CT demonstrates limited utility in the context of SRC beyond detecting craniofacial fractures or surgical emergencies, reinforcing clinical assessment as the diagnostic cornerstone. Protocol optimisation should prioritise radiation risk mitigation through strict adherence to paediatric low-dose guidelines.

**Supplementary Information:**

The online version contains supplementary material available at 10.1007/s40279-025-02195-5.

## Key Points

**Table:** 

The commonest CT-evident rugby-related head injuries included craniofacial fractures, cerebral haemorrhage, contusions and oedema.
Despite its frequent use in the assessment of rugby-related head injuries, CT imaging has low sensitivity and negative predictive value.
Future longitudinal studies should investigate the correlation between advanced imaging, biomarkers and metrics of head impact load to aid in the development of assessment methodologies that are more sensitive to the changes of sport-related concussion.

## Introduction

### General Background

Globally, traumatic brain injury (TBI) affects 50 million people annually, imposing a $400 billion economic burden, predominantly in low- to middle-income countries (LMIC) [1]. In the USA, 1.7–3.8 million sports-related concussions (SRC) occur yearly [2], constituting 5–9% of all sports related injuries [3]. From 2001 to 2009, emergency visits for SRCs among individuals aged 19 years and younger rose by 6.2% annually, comprising 30% of all concussions in this age group [3, 4].

Research increasingly associates concussion and repetitive head impacts (RHI) with adverse long-term neurocognitive outcomes [5–10]. Furthermore, physiological changes in the brain may persist beyond clinical recovery, complicating treatment [11–13]. Most concussions recover clinically within 10–14 days [13]; yet, a recent study found that over half of participants had not fully recovered after 2 weeks, and a significant number remained symptomatic after 4 weeks [14]. One of the largest longitudinal studies investigating collegiate SRC found that natural recovery occurred in most athletes only at 28 days [15].

### Rugby Union

Rugby Union (rugby) is a popular team-based sport played by 9.1 million registered players in 121 countries around the world. In South Africa, there are 603,455 players, of whom 530,393 are officially registered [16].

Rugby, a physically demanding collision sport, involves deliberate player contact, unlike contact sports where contact is non-deliberate, e.g. basketball and soccer [17].

### Sport-Related Concussion (SRC): Definition and Impact

SRC is defined as ‘a traumatic sporting-related brain injury induced by biomechanical forces caused either by a direct blow to the head, face, neck or elsewhere on the body with an impulsive force transmitted to the head’. Symptoms can vary in type and severity and are not attributable to other causes, e.g. drugs or other injuries. Standard structural neuroimaging is normal [18].

SRC significantly burdens healthcare, academics and quality of life for youth athletes [19–22]. Estimates suggest that reducing injuries in contact sports could prevent approximately 6900 SRCs annually and save between $500 million and $1.5 billion in healthcare costs [23]. In addition, many student-athletes face psychological, physical and academic challenges post-SRC, with data indicating that 33–50% do not report their symptoms promptly [24–26].

### Rugby Union: Injury Burden

A recent systematic review reported an injury incidence of 40 and 69 per 1000 player hours and 6 and 34 SRCs per 1000 player hours in male and female youth rugby, respectively [27]. An English school-level rugby surveillance study found an injury incidence of 31 per 1000 player hours and an SRC incidence of 9 per 1000 player hours. Head and neck injuries accounted for 62% of all injuries at the under-15 level, and 38% and 34% at under-13 and under-18 levels, respectively. SRC comprised 30% of all injuries [28].

Leahy et al. [29] reported 54 injuries per 1000 player hours in Irish school rugby. SRC was more common in forwards (rate ratio 3.5). A study in under-15 players found craniofacial injuries most prevalent, with SRC representing 38% of all injuries [30].

The South African Rugby Union Youth Week Injury Surveillance Report 2019 [31] reported 18 injuries and 3 SRCs per 1000 player hours. Head and neck injuries were most common among youth players aged 13–18 years over six seasons of annual elite-level youth tournament weeks [32]. A 1-year study of a South African high school rugby team reported 34 injuries and 13 SRCs per 1000 player hours, with head, neck, and spine injuries comprising 38% of all injuries [33].

### Imaging in the Context of Concussion

The latest Concussion in Sport Group consensus now recognises concussion may cause axonal injury, blood flow disturbances and neuronal inflammation [18].

Computed tomography (CT) is commonly used in concussion and SRC assessment [34]. There are no data regarding the referral pattern for imaging following rugby-related head injuries; nonetheless, a geographically pertinent study from a tertiary referral centre in the Western Cape reported that approximately a third of 522 patients referred for imaging of possible traumatic brain injury had abnormal CT imaging [35].

A landmark 2009 US study found TBI^1^ in 5.2% of 14,969 patients [36]. A secondary analysis of the study identified 3289 children who sustained sport-related head injuries. CT was performed in the majority, with TBI evident in 4% [37]. Rose et al. [38] identified 1953 participants with SRC of whom 10% (*n* = 193) underwent CT imaging. TBI was evident in 3.1% of CT scans.

CT contributes 50% of the US population’s cumulative radiation dose, despite comprising only 12% of radiological studies. Its use in paediatrics has increased tenfold since 1980, with a 10% annual rise [39]. A US study of 750 million emergency visits, including 4 million for mild traumatic brain injury (mTBI), reported a 36% increase in CT use for mTBI from 2006 to 2011, despite a reduction in injury severity [40].

The potential risks of ionising radiation are widely recognised. Children are more vulnerable to ionising radiation owing to their longer period for expression of radiation effects and potential exposure to higher doses if CT parameters are not adjusted for their smaller size [39].

Large-scale studies have shown long-term effects of radiation exposure. The European EPI-CT trial found a linear dose–response relationship between radiation exposure and brain cancer risk in over 650,000 participants [41]. An Australian study observed a 16% increased cancer risk per additional CT scan and a 24% higher lifetime cancer risk in participants exposed to radiation compared with unexposed controls. Early exposure was associated with increased risk [42].

Early exposure to ionising radiation is associated with an increased risk of developing cancer later in life owing to a combination of biological and physiological factors. Children are particularly vulnerable because their cells are still developing and dividing rapidly, making them more susceptible to DNA damage caused by radiation. This increases the likelihood that radiation-induced mutations will persist and potentially lead to malignancies over time.

In addition, children absorb a relatively higher radiation dose than adults for the same CT scan owing to their smaller body size and differences in tissue composition. This higher relative dose results in greater exposure of developing tissues to ionising radiation, thereby amplifying the potential for DNA damage.

Furthermore, radiation-induced cancers often have a long latency period, typically taking decades to manifest. Given their longer remaining lifespan, children have a greater window of time for radiation-induced genetic mutations and epigenetic changes to accumulate, increasing their lifetime risk of developing malignancies.

Recent imaging advancements, e.g. diffusion tensor imaging (DTI) and magnetic resonance imaging (MRI) with arterial spin labelling (ASL) or time-of-flight angiography, consistently demonstrate structural brain changes following concussion and RHIs, even in the absence of concussion [43–52]. Studies using advanced MRI techniques to investigate SRC and RHIs have been conducted in affluent First World countries [53]. These advanced modalities can detect subtle microstructural changes and functional abnormalities in the brain that are often undetectable with CT, providing a more comprehensive understanding of the injury. For instance, DTI can reveal axonal injury and changes in white matter integrity, while ASL can assess cerebral blood flow alterations associated with concussion. Importantly, MRI does not produce ionising radiation. However, these advanced imaging techniques also have limitations. They are often more expensive and less accessible than CT, particularly in LMICs, which may restrict their widespread use in clinical settings. In addition, advanced imaging requires longer acquisition times and may not be suitable for acute care scenarios where rapid decision-making is crucial. Furthermore, the interpretation of these advanced imaging findings can be complex and may require specialized expertise that is not always available in all healthcare facilities.

### Burden of Paediatric Concussion

Concussion significantly affects children and adolescents, impacting schooling, social interaction, family dynamics, sports participation and mental health [54]. Concussed adolescents often perform worse on neuropsychological tests [55], with cognitive impairment lasting up to 6 months post-injury [56]. A South African study indicated that adolescents without SRC showed greater academic improvement than those with multiple SRCs [57]. A scoping review of 14 studies found that SRC negatively impacts academic achievement [58]. In Australia, children hospitalised for SRC were less likely to meet literacy and numeracy standards and more likely to leave school early compared with their peers [59].

Adolescent football players exhibit structural brain changes, such as cortical thinning and increased sulcation in key brain regions. These abnormalities may persist for up to 4 months post-SRC [49, 60]. Ongoing microstructural reorganisation and neuroinflammation can persist for up to 3 months [50]. Adolescent ice hockey players displayed white matter abnormalities 3 months post-injury [61]. These findings raise concerns about structural axonal injuries in the still-maturing adolescent brain, with sub-concussive impacts also linked to blood–brain barrier disruption [62].

To our knowledge, no neuro-imaging studies of school-level rugby players in an LMIC have been reported.

Therefore, we aim to report the imaging findings of school-level players in a LMIC who presented to a public tertiary referral centre or a private radiology service provider for imaging after sustaining a traumatic rugby-related head injury and examine (1) the prevalence of concussion based on available clinical history; (2) the imaging correlation of players with concussion and the diagnostic utility of CT in the context of concussion; and (3) compare the imaging findings between the public and private sector.

## Methods

### Study Design

The study is designed as a descriptive, retrospective cross-sectional correlational study spanning a 10-year period from 2014 to 2023.

### Setting and Study Population

The study was conducted in the Western Cape region of South Africa with an estimated population of 7.5 million.^2^

Multi-centre data collection occurred at Tygerberg Hospital, a large 1400-bed tertiary referral centre serving the Cape Northern and Eastern Metro regions, as well as the Cape Winelands, rural Overberg and West Coast districts. It is one of three tertiary referral centres in the province, where over 80% of the population lacks medical insurance and relies on public health services [63].

Data from the private sector were collected at SCP Radiology, a large multi-centre private radiology provider with a geographic presence similar to that of Tygerberg Hospital. SCP has branches near Tygerberg Hospital and in corresponding district regions, including Paarl, Worcester, Vredenburg and Vredendal.

### Participants

Participants included school-aged players (6–18 years) who presented to or were referred to either Tygerberg Hospital or SCP Radiology for imaging of rugby-related head injuries.

### Data Collection

The data-mining capabilities of the radiological information system (RIS) at each institution were utilised to conduct a filtered search for the required data. Searches focused on clinical histories and radiological reports containing the term ‘rugby’, restricted to ‘head’ or ‘brain’ studies. Each case was reviewed to ensure inclusion of only rugby-related head injuries. Data extracted included participant age, imaging modality, study type, study date and SRC presence based on clinical information.

### Measures

Collected independent variables comprised participant age, imaging modality, study type and findings; the dependent variable was SRC diagnosis (yes or no). The study date was recorded as a contextual variable.

SRC diagnoses were recorded only when signs or symptoms included in the clinical history of the imaging request, as provided by the referring clinician, met the criteria outlined in the most recent Sport Concussion Assessment Tool 6 (Supplementary 1) [18, 64]. Injury recording adhered to the latest International Olympic Committee consensus recommendations [65].

Descriptive statistics were performed to analyse the data using Microsoft Excel and R [66]. Quantitative variables included participant age (mean ± SD) and SRC status (yes or no), while qualitative variables encompassed imaging findings categorised by injury type. Descriptive statistics were performed to calculate means, standard deviations and frequencies. Proportions were calculated to investigate imaging trends in the comparative statistical analysis between the public and private datasets. The normality of participant age was assessed using the Shapiro–Wilk test, which did not indicate significant deviation from normality (*p* > 0.05). Logistic regression analysis and a Fisher’s exact test (with 95% confidence intervals (Cis) and significance set at *p* < 0.05) were conducted to analyse the binary outcome of SRC occurrence across sectors and to compare the prevalence of abnormal CT findings between concussed and non-concussed players.

The clinical utility of CT was estimated through the calculation of sensitivity and specificity and positive and negative predictive values (see Limitations). True or false positives and negatives were defined as follows:

- true positive: abnormal imaging when SRC clinical criteria were met;
- false positive: abnormal imaging when SRC clinical criteria were not met;
- true negative: normal imaging when SRC clinical criteria were not met;
- false negative: normal imaging when SRC clinical criteria were met.

Imaging findings were deemed to be abnormal when any of the following features were present: intracranial haemorrhage or contusion, cerebral oedema, traumatic infarction, diffuse axonal injury, sigmoid sinus thrombosis, midline shift of intracranial contents or signs of brain herniation, skull diastasis, pneumocephalus, or skull fracture.

### Definitions

Concussion, often termed mild traumatic brain injury (mTBI) in North American literature, refers to an injury from any cause; SRC specifically denotes an injury resulting from sports participation [37].

The terms concussion and mTBI are frequently used interchangeably, although this is still debated by some authors [54]. Notwithstanding, the American Congress of Rehabilitation Medicine states that the terms concussion and mTBI may be used interchangeably when neuroimaging is normal or not clinically indicated [67].

TBI in the context of CT imaging includes intracranial haemorrhage, cerebral contusion or oedema, diffuse axonal injury, sigmoid sinus thrombosis, midline shift of intracranial contents, signs of brain herniation, skull suture diastasis and fractures, etc [36].

### Sample Size

Sample size calculations for this study were based on power analysis for logistic regression owing to the binary nature of the outcome variable (SRC occurrence). The analysis aimed to ensure sufficient power to detect clinically relevant differences in SRC odds between comparison groups.

Required sample sizes were calculated using the pwr package in R with arcsine transformation for proportion comparisons in binomial distributions [66]. The specific formula used for converting odds ratios (ORs) to effect sizes is $h = \log \left( {{\text{OR}}} \right)/\sqrt {3.29}$, facilitating the use of the arcsine difference method for power calculations.

Assuming a baseline SRC probability of 10%, a significance level of 0.05 and 80% power to detect specified effect sizes: 157 participants were needed for a small effect size (OR = 1.5), 31 for a moderate effect size (OR = 2.5) and 12 for a large effect size (OR = 4.5).

### Data Management

Data collection was fully anonymised; no patient identifiers were recorded or exported from the RIS. Anonymised data were stored on a password-protected computer belonging to the principal investigator.

### Ethical Considerations

Ethical approval was granted by the Health Research Ethics Committee of Stellenbosch University (reference no. S24/03/076). Imaging data were collected retrospectively in an anonymised manner that precludes tracing back to specific participants. The study was conducted in accordance with the Declaration of Helsinki.

## Results

Overall, 369 school-level participants referred for imaging of rugby-related head injury, with a mean (± standard deviation, SD) age of 15 (± 2.5) years, were included in the study (Table 1).

**Table 1:** Participant age distribution and SRC proportions

	Overall (*n* = 369)	Private (*n* = 237)	Public (*n* = 132)
Median age (years; IQR)	15 (13–17)	15 (13–17)	16 (14–17)
SRC (%)	289 (78)	164 (69)	125 (95)
No SRC (%)	80 (22)	73 (31)	7 (5)

*SRC* sport-related concussion, *SD* standard deviation, *IQR* interquartile range, *n* count, *%* proportion

### Concussion Prevalence and Imaging Studies

The overall proportion of participants with SRC based on clinical criteria [18] was 78% (Table 1).

Unadjusted logistic regression modelling showed that the odds ratio (OR) for SRC in the public sector compared with the private sector was 8.39 (95% CI 8.37–8.41, *p* < 0.001), i.e. participants in the public sector were over eight times more likely to have an SRC, holding all other variables constant.

CT was the primary imaging modality (Table 2), comprising 347 (94%) of the imaging studies performed across both sectors. Uncontrasted CT of the brain (*n* = 269) and CT of the brain and cervical spine (*n* = 53) were the most frequent study types.

**Table 2:** Imaging modalities by sector

	CT	MRI
Sector		
Overall	347	22
Private	217	20
Public	130	2

*CT* computed tomography, *MRI* magnetic resonance imaging

In total, 21 MRI studies were normal, with 1 abnormal study in the private sector not related to trauma (multifocal leuco-encephalopathy). In addition, 17 participants with SRC had normal MRI imaging.

### Statistical Analysis of Head Injuries

Of the 369 studies performed, 319 (86%) studies were normal (Table 3). The most frequent findings in the 50 (14%) abnormal studies comprised craniofacial fractures and brain injuries (Table 3).

**Table 3:** Between-sector comparison of imaging injury categories

	Overall (*n* = 369)	Private (*n* = 237)	Public (*n* = 132)
Injury category			
No injury (%)	319 (86)	219 (92)	100 (76)
Fracture (%)	28 (8)	9 (4)	19 (14)
Brain injury (%)	19 (5)	9 (4)	10 (8)
Soft tissue (%)	3 (1)	0 (0)	3 (2)

*n* count, *%* proportion

**Fig. 1:**
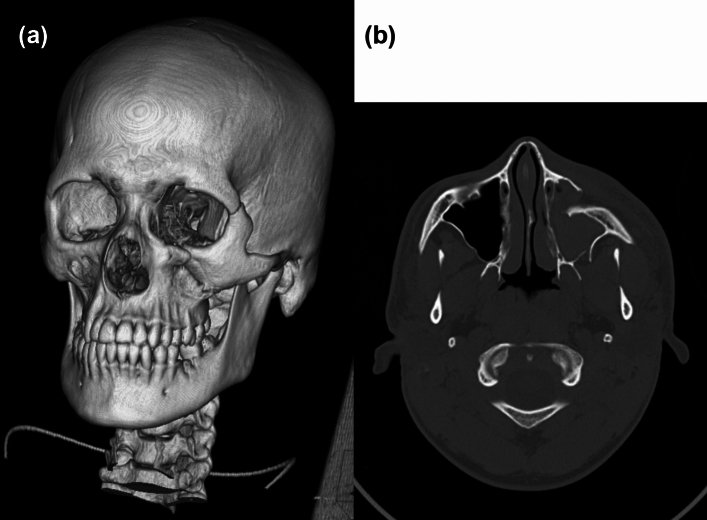
Three-dimensional volume rendering (**a**) and axial bone window CT image (**b**) of a 17-year-old player with a markedly displaced left-sided zygomatico-maxillary complex fracture

**Fig. 2:**
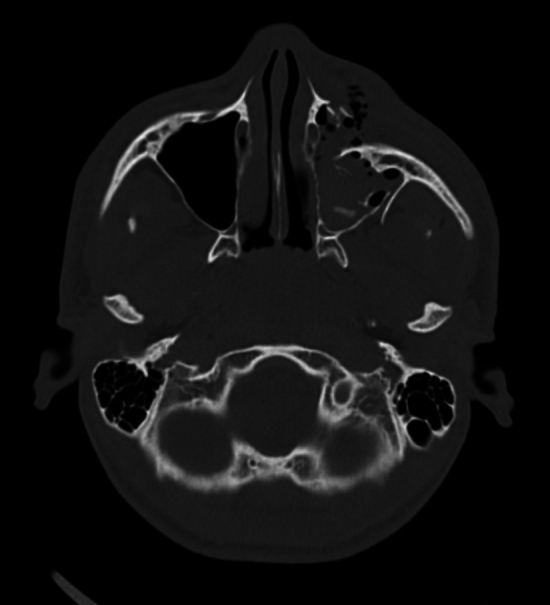
Axial bone window CT image of a 16-year-old player with a displaced left zygomatico-maxillary complex fracture

Craniofacial fractures included fractures of the frontal and parietal bones, in addition to orbit, zygoma, maxilla, mandible, and nasal bone fractures. Illustrative cases are shown in Figs. 1 and 2.

**Fig. 3:**
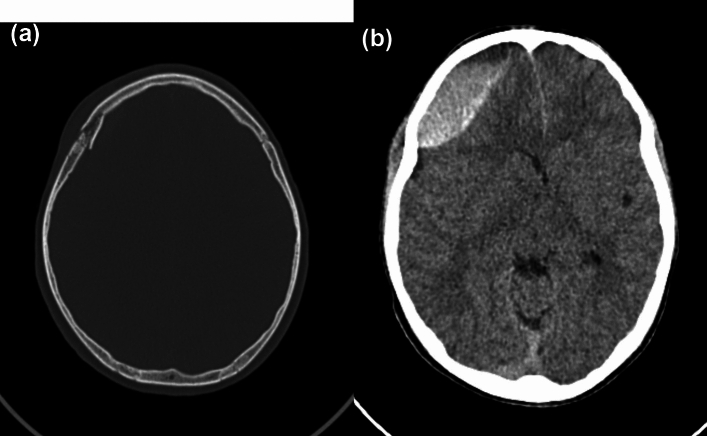
A 12-year-old player presented with a Glasgow coma scale (GCS) of 13/15. Axial CT demonstrated a comminuted, depressed fracture of the right fronto-parietal bone (**a**) with a large underlying extradural haematoma causing mass effect with 5 mm of midline shift to the left and subfalcine herniation (**b**)

**Fig. 4:**
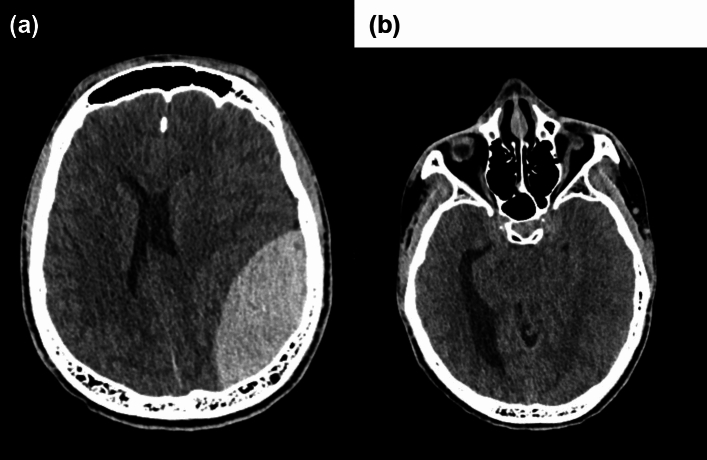
**a** and **b** 16-year-old player presented with deteriorating level of consciousness with a GCS of 7/15 at initial presentation. Axial CT imaging demonstrated a large left extradural haematoma (41 mm in depth), 8 mm midline shift, subfalcine and uncal herniation, and contralateral entrapment hydrocephalus

Intracranial injuries included intra- and extra-axial haemorrhage, cerebral oedema and cerebral contusion. One participant had subfalcine and uncal herniation. Illustrative cases are shown in Figs. 3 and 4.

### Diagnostic Utility of CT in the Context of SRC

CT was performed in 347 cases (94%), with abnormal findings reported in 50 studies (14%; private *n* = 18, public *n* = 32).

CT had a positive predictive value (PPV) of 78%, negative predictive value (NPV) of 22%, sensitivity of 14% and specificity of 85% (Table 4).

**Table 4:** Sensitivity, specificity, and negative and positive predictive values of CT in SRC

	Concussed	Non-concussed		
Imaging				
Abnormal	39	11	PPV	78%
Normal	233	64	NPV	22%
	Sensitivity 14%	Specificity 85%		

*SRC* sport-related concussion, *PPV* positive predictive value, *NPV* negative predictive value

The proportion of abnormal versus normal CTs was similar in concussed and non-concussed players (± 17%). Fisher’s exact test indicated no statistically significant difference in abnormal CT findings between the groups (OR = 0.97; 95% CI 0.46–2.23; *p* = 1.0), suggesting no association between concussion status and abnormal CT results.

## Discussion

Over the 10-year study period, 369 school-level rugby players underwent imaging following head injuries. Uncontrasted CT was used most frequently (*n* = 347, 94%), with 50 (14%) showing trauma-related abnormalities, including craniofacial fractures and cerebral oedema or contusions.

Based on referring clinicians’ clinical information provided in imaging requests, the overall SRC prevalence was 78% (*n* = 289), with notable sector differences: 69% in the private and 95% in the public sector, where participants were 8.39 times more likely to have an SRC. Among those with normal CT (*n* = 297), 233 (78%) had an SRC, yielding a sensitivity of 14% and NPV of 22%.

### Imaging Findings and Clinical Implications

In our study, CT identified abnormalities in only 14% of participants, raising concerns about its utility in the context of SRC, as most cases lacked detectable structural changes. The lack of significant differences in abnormal CT findings between concussed and non-concussed players indicates that concussion status may not predict structural abnormalities in this cohort. This is consistent with literature highlighting CT’s low sensitivity for detecting sport-related concussion, as it primarily reveals gross structural changes, such as fractures and haemorrhages, rather than the microstructural and physiological alterations typical of SRC. The odds ratio of 0.97 (95% CI 0.46–2.23) underscores the variability and lack of association between CT findings and concussion status, reinforcing that CT alone is insufficient for SRC diagnosis.

Nonetheless, CT is frequently employed in the initial assessment of paediatric mild traumatic brain injury (pmTBI) and SRC. In a large 2009 US study by Kuppermann et al., [36] a total of 42,412 children with a mean age of 7 (± 6) years were enrolled across 25 paediatric emergency departments, focussing on those presenting within 24 h of head trauma. CT was performed on 14,969 (35.3%) children. TBIs were found in 780 (5.2%) children. Clinically important TBIs (ciTBIs) were detected in 376 (0.9%) children, of whom 60 (0.1%) required neurosurgery. Prediction rules were applied to reduce the number of CT studies performed. In children older than 2 years of age, the rules included normal mental status, absence of scalp hematoma (except frontal) and no loss of consciousness longer than 5 s. This rule demonstrated an NPV of 100% and a sensitivity of 100%. The validated prediction rules effectively identified children at very low risk for ciTBIs, suggesting that many could safely forgo CT imaging. Neither rule missed cases requiring neurosurgery in the validation populations. This study highlights the potential for improved decision-making in emergency settings regarding paediatric head trauma management.

A secondary analysis of the Kuppermann study, focussing specifically on sport-related head injuries, was performed by Glass et al. [37] Of the initial cohort of over 42,000 participants, 3289 (14%) of 23,082 children aged 5–18 years sustained sport-related blunt head trauma. Median ages ranged from 11 to 16 years, depending on the sport category. CT imaging was performed in 1743 (53%) of the 3289 children, with only 69 (4%) TBIs detected.

Ellis et al. [68] reviewed the medical records of patients who presented to a Canadian multidisciplinary paediatric SRC programme. The authors investigated the frequency of neuroimaging (CT and MRI) and the findings of imaging studies performed. In total, 36 (24%) of 151 patients with a mean age of 14 years underwent imaging, which was normal in 78%. CT imaging was normal in 19 (79%) of 24 studies performed. The authors concluded that neuroimaging studies are normal in most patients with SRC.

A similar study was performed by Rose et al. [38] at a large US children’s hospital. Of 1953 patients with a mean age of 14 years who presented to the SRC clinic, 193 CT and 134 MRI studies were performed. The authors reported that only 3.1% of CT studies and 1.5% of MRI studies demonstrated signs of traumatic brain injury.

In our study, the proportion of TBIs detected on imaging in children of similar ages to those reported above was 14%. This is lower than the proportion reported by Ellis but three to four times higher than those reported by Kupperman, Glass, and Rose. It should be noted that, unlike the studies mentioned above, all participants in our study underwent imaging, i.e. there is no subset of participants who did not receive imaging. Patients referred for imaging are likely to have more severe injuries than those not referred for imaging, making abnormal neuroimaging results more probable compared with the general paediatric SRC population. Although Ellis reported that TBI was evident in 21% of cases, only 24 CT scans were performed, meaning the findings may have been skewed by the small sample size. Speculatively, the proportion of CT studies with evident TBIs in our study may have been lower had it not been for the selective nature of the study cohort.

Notwithstanding the comparatively high proportion of TBIs, our findings affirm that a substantial proportion of participants subjected to head impacts in sport, rugby in particular, undergo CT imaging with a minority demonstrating TBIs on imaging.

Given that concussion is now recognised as a complex microstructural injury involving physiological and biochemical disturbances, it is essential to maintain a high index of suspicion and explore alternative imaging modalities [53, 69]. Advanced techniques, such as DTI and magnetic resonance (MR) angiography, can provide deeper insights into microstructural changes and cerebral blood flow alterations associated with concussions and may enhance our understanding of the long-term effects of head injuries, particularly in developing adolescent brains. Collectively, these findings highlight the limitations of CT in paediatric SRC assessment, emphasising the need for advanced imaging techniques that are more sensitive to SRC-related changes. The results further emphasise the need for the development of imaging protocols that balance the limited diagnostic value of CT with the associated radiation risks, especially in paediatric populations.

### Prevalence and Context of SRC

The high prevalence of sport-related concussion (SRC) observed in this study is consistent with existing literature that highlights children’s vulnerability to head injuries in contact sports, particularly youth rugby players, who are at an increased risk owing to frequent high-impact collisions [27, 70].

The observed differences in SRC prevalence between public and private sector participants may suggest potential issues related to resource access, coaching quality and injury management protocols that could be influenced by socioeconomic factors. It is possible that public sector players experience increased exposure to concussive impacts and may have less effective injury prevention strategies, which could be compounded by the challenges of a resource-limited healthcare system. Most individuals in the Western Cape depend on public health services [63], which raises the clinical threshold for referrals to tertiary centres for specialised care. Speculatively, it is likely that the cohort referred for imaging in the public sector had higher injury severity owing to this elevated threshold.

Furthermore, the high public sector SRC prevalence may stem from differences in playing conditions, access to protective gear, intensity of play, and variability in training and adherence to safety protocols. Thus, interventions to reduce concussion rates should be context-specific, emphasising a systems approach and the importance of high-quality coaching and proper tackle technique training for improved player safety [71–73].

### Integration of Advanced Imaging and Biomarker Research

Our findings align with recent advancements in neuroimaging and biomarker research, highlighting opportunities for the early detection and monitoring of brain health in athletes subjected to repetitive head impacts.

Techniques such as DTI and ASL have markedly improved the detection of microstructural and functional changes in the brain. If incorporated into routine clinical practice, currently limited by high study costs and lengthy study times influencing paediatric studies, these techniques could enhance SRC management [53, 69].

Nonetheless, MRI technological advancement, demonstrated by the recent commissioning of an 11.7 Tesla scanner, may ensure that access to advanced imaging becomes more readily accessible in the not-too-distant future [74].

Furthermore, the development of serum biomarkers such as S100B, GFAP and UCH-L1 provides non-invasive assessment of brain injury severity and could potentially guide decisions related to the necessity of imaging studies such as CT, thereby reducing unnecessary radiation exposure [51, 75].

### Future Directions

The findings lay a groundwork for further investigation in SRC imaging in youth rugby in LMICs.

Key research areas include:

- Referral patterns and post-imaging management: Exploring referral patterns for imaging in suspected SRC cases and the clinical outcomes of patients with positive imaging findings to better understand the implications of imaging practices on head injury management in youth rugby players.
- Longitudinal studies: Tracking recovery and long-term neurocognitive effects on young athletes using advanced neuroimaging and biomarkers to understand brain changes and evaluate interventions, incorporating exposure data to determine injury incidence rates.
- Advanced imaging techniques: Investigating neuroimaging methods that detect subtle brain changes not visible on conventional CT.
- Intervention strategies: Evaluating rule changes and educational programmes aimed at reducing SRC incidence among youth athletes.

### Study Limitations

The retrospective design, while enabling a broad 10-year analysis, is subject to biases inherent in historical data collection and interpretation.

Underreporting of SRC, particularly in settings where there is poor stakeholder education and baseline SRC awareness owing to limited medical oversight, may further affect the accuracy of prevalence rates.

The absence of exposure data, although not the focus of this study, prevents the calculation of SRC incidence. In addition, reliance on CT alone may not fully capture the spectrum of SRC severity. This limitation arises because CT imaging is very effective at identifying structural abnormalities, such as fractures or significant intracranial haemorrhage, while neurophysiological and microstructural changes associated with concussions often remain undetectable on CT scans. Consequently, it is possible that some cases of SRC may be overlooked or mischaracterised when solely relying on CT findings, as this imaging technique does not adequately reflect the functional impairments or subtle microstructural brain changes that can occur in concussion.

Selection bias is introduced by the referral process, where patients are sent for imaging after first-contact evaluation, typically on the basis of clinical justification, such as visible craniofacial or suspected occult intracranial injuries following head trauma. Thus, it is probable that most players that were referred had at least an SRC, or a more severe injury warranting imaging, though the confirmation of SRC relied on clinical histories documented in imaging study requests by referring clinicians, which may not always have been sufficient.

Few cases of head injuries are referred for imaging in rugby. As noted, head and neck injuries, particularly SRC, are common in school rugby [27, 31]. For players under 18 years old, an SRC incidence of 10 per 1000 player hours suggests one SRC every 3.3 matches. On a typical school sports day with 8–20 matches, 3–6 SRCs could occur, with up to half going unreported.

## Conclusions

This study is the first to report imaging findings of head injuries in school-level rugby, highlighting their prevalence and characteristics. The most common CT-evident injuries included craniofacial fractures, cerebral haemorrhage, contusions and oedema.

Despite the anticipated high prevalence of SRC, CT demonstrates low sensitivity for diagnosis. The absence of significant differences in abnormal CT findings between concussed and non-concussed players indicates that CT alone is insufficient for SRC diagnosis, reinforcing the need for advanced imaging techniques. Advanced imaging techniques and biomarkers offer valuable resources for improving the accuracy of SRC diagnoses.

Differences in SRC rates between public and private sectors reflect disparities in healthcare access and injury reporting. Educational initiatives are essential to raise awareness of SRC symptoms among athletes, coaches, parents and clinicians.

Future research should use advanced imaging and biomarkers for a more complete assessment of brain health in young athletes, improving protection against the serious effects of sport-related head injuries.

As our understanding of SRC’s long-term impacts evolves, prevention and management strategies must also adapt. Evidence-based approaches tailored to specific sports contexts will help reduce the risk of traumatic brain injuries in youth athletes.

## Supplementary Information

Below is the link to the electronic supplementary material.

Supplementary file1 (PDF 471 KB)

## Footnotes

1 Defined as intracranial haemorrhage or contusion, cerebral oedema, traumatic infarction, diffuse axonal injury, sigmoid sinus thrombosis, midline shift of intracranial contents or signs of brain herniation, skull diastasis, pneumocephalus or skull fracture depressed by at least the width of the skull table.

2 https://census.statssa.gov.za/assets/documents/2022/P03014_Census_2022_Statistical_Release.pdf.

## Declarations

### Availability of data and material

The data that support the findings of this study are available upon reasonable request from the corresponding author, R.v.T.
