# Supplementary material for: Radiological Correlates of Head Injuries in School-Level Rugby Union: A 10-Year Retrospective Cross-Sectional Analysis
Source: Sports Med. 2025 Mar 25;55(7):1783–95. doi: 10.1007/s40279-025-02195-5 (PMC12296969; doi:10.1007/s40279-025-02195-5)
Supplement: Supplementary file 1 — Supplementary file1 (PDF 471 KB) [file 40279_2025_2195_MOESM1_ESM.pdf]

Radiological correlates of head injuries in school-level rugby union: a 10-year retrospective cross-sectional analysis

Corresponding author

Riaan van Tonder Division of Radiodiagnosis, Stellenbosch University

13139614@sun.ac.za

ORCID 0000-0003-2858-0863

Co-authors

**Dr Hofmeyr Viljoen**	SCP Radiology, Cape Town, ZA
**Prof Christelle Ackermann**<br>**ORCID 0000-0002-4598-1127**	Division of Radiodiagnosis, Stellenbosch University, Cape Town, ZA

Journal

Sports Medicine

Supplementary 1

Criteria used to assess the presence or absence of sport-related concussion (SRC)

SRC diagnoses were recorded only when the captured clinical history or radiological report included signs or symptoms considered diagnostic of SRC, as outlined in the most recent concussion consensus statement in "Box 1: Red Flags" or "Step 1: Observable Signs" of the Sport Concussion Assessment Tool 6.(17, 62)

Common red flags encountered include loss of consciousness, severe or worsening headache, deteriorating consciousness, Glasgow Coma Scale <15, vomiting, and visible skull deformity. Observable signs include lying motionless, falling unprotected, ataxia, confusion, blank or vacant look, and visible facial injury.

Step 1: Observable Signs

Witnessed ☐ Observed on Video ☐

Lying motionless on playing surface	Y	N
Falling unprotected to the surface	Y	N
Balance/gait difficulties, motor incoordination, ataxia: stumbling, slow/ laboured movements	Y	N
Disorientation or confusion, staring or limited responsiveness, or an inability to respond appropriately to questions	Y	N
Blank or vacant look	Y	N
Facial injury after head trauma	Y	N
Impact seizure	Y	N
High-risk mechanism of injury (sport-dependent)	Y	N

Step 2: Glasgow Coma Scale

Typically, GCS is assessed once. Additional scoring columns are provided for monitoring over time, if needed.

Time of Assessment:

Date of Assessment:

Best Eye Response (E)			
No eye opening	1	1	1
Eye opening to pain	2	2	2
Eye opening to speech	3	3	3
Eyes opening spontaneously	4	4	4

Best Verbal Response (V)			
No verbal response	1	1	1
Incomprehensible sounds	2	2	2
Inappropriate words	3	3	3
Confused	4	4	4
Oriented	5	5	5

Best Motor Response (V)			
No motor response	1	1	1
Extension to pain	2	2	2
Abnormal flexion to pain	3	3	3
Flexion/withdrawal to pain	4	4	4
Localized to pain	5	5	5
Obeys commands	6	6	6

Glasgow Coma Score (E + V + M)			

Box 1: Red Flags

- Neck pain or tenderness
- Seizure or convulsion
- Double vision
- Loss of consciousness
- Weakness or tingling/burning in more than 1 arm or in the legs
- Deteriorating conscious state
- Vomiting
- Severe or increasing headache
- Increasingly restless, agitated or combative
- GCS <15
- Visible deformity of the skull

Step 3: Cervical Spine Assessment

In a patient who is not lucid or fully conscious, a cervical spine injury should be assumed and spinal precautions taken.

Does the athlete report neck pain at rest?	Y	N
Is there tenderness to palpation?	Y	N
If NO neck pain and NO tenderness, does the athlete have a full range of ACTIVE pain free movement?	Y	N
Are limb strength and sensation normal?	Y	N

Step 4: Coordination & Ocular/Motor Screen

Coordination: Is finger-to-nose normal for both hands with eyes open and closed?	Y	N
Ocular/Motor: Without moving their head or neck, can the patient look side-to-side and up-and-down without double vision?	Y	N
Are observed extraocular eye movements normal? If not, describe:	Y	N

Step 5: Memory Assessment Maddocks Questions[1]

Say ***"I am going to ask you a few questions, please listen carefully and give your best effort. First, tell me what happened?"***

Modified Maddocks questions (Modified appropriately for each sport; 1 point for each correct answer)

What venue are we at today?	0	1
Which half is it now?	0	1
Who scored last in this match?	0	1
What team did you play last week/game?	0	1
Did your team win the last game?	0	1
**Maddocks Score**		/5

Note: Appropriate sport-specific questions may be substituted

For use by Health Care Professionals only

British Journal of Sports Medicine
